# Emotional Tension as a Frame for Argumentation and Decision-Making: Vegetarian vs. Omnivorous Diets

**DOI:** 10.3389/fpsyg.2021.662141

**Published:** 2021-06-08

**Authors:** María Pilar Jiménez-Aleixandre, Pablo Brocos

**Affiliations:** Departamento de Didácticas Aplicadas, Universidade de Santiago de Compostela, Santiago de Compostela, Spain

**Keywords:** argumentation, emotions, discourse, decision-making, vegetarianism, sustainable diet

## Abstract

Argumentative discourse has a complexity that is not entirely captured by purely structural analyses. In arguments about socio-scientific issues (SSI), a range of dimensions, besides scientific knowledge, including values, ethical concerns, cultural habits, or emotions, are mobilized. The relationship between argumentation and emotions is now drawing attention of researchers. Our focus is on the dynamic interactions among emotions and scientific evidence. We draw from Plantin, who proposed that emotions are mobilized as argumentative resources alongside knowledge. The goal of our study is to examine in which ways emotional tension frames the construction of arguments about vegetarian vs. omnivorous diets (ODs) with a group of four preservice teachers. The results suggest that the interactions between the group emotional tension and the evaluation of evidence drive a change toward a decision that would be emotionally acceptable for all participants. Participants attended to the epistemic dimension, weighing evidence, and values about the choices, but the emotional framing took priority. We suggest that the analysis of this emotive framing may be a fruitful approach for sophisticated studies of argumentation beyond structural issues.

## Introduction: Argumentation and Emotions About Diets

The analysis of the structure of arguments—in other words, the number, quality, and relationships between components such as claims, data, justifications, or rebuttals—has yielded relevant insights about how knowledge is justified or, more generally, evaluated (e.g., Berland and McNeill, 2010; Osborne et al., 2016; Bravo-Torija and Jiménez-Aleixandre, 2018). Argumentative discourse has, however, a complexity that is not entirely captured by purely structural analyses. In particular, in arguments about socio-scientific issues (SSI), a range of dimensions, besides scientific knowledge, including values, ethical concerns, cultural habits, or emotions, are mobilized. The relationship between argumentation and emotions, understudied for years, is recently drawing attention of researchers (e.g., Micheli, 2010; Baker et al., 2013a; Pollaroli et al., 2019). Previous work on argumentation about SSI addressed this relationship in terms of distinctions among types of arguments, for instance, Zeidler and Sadler's (2008) description of three argumentative patterns: rational, intuitive, and emotive. This study is framed in a different perspective, for we are interested in the dynamic interactions among emotions, evaluation of scientific evidence, and other dimensions such as cultural identities or ethical concerns, what for Hufnagel (2015, 2019, 2021) constitutes emotional sense-making. Plantin (2011) took a novel approach to the relationships between argumentation and emotions, conceiving emotions as argumentative resources that are mobilized alongside knowledge. We draw from Plantin's (2019) work about variation of interactional tension as a defining feature of emotions in discourse, combining it with Andriessen et al.'s (2011) approach to socio-cognitive tension in argumentative interactions. The balance between engagement in argumentation and sustaining favorable socio-emotional processes has been explored by Isohätälä et al. (2018). By socio-emotional processes, they mean the social and emotional dimensions of collaborative learning: social “in the sense that they are dynamically created within the interpersonal setting through the social interactions that the learners engage in” (p. 2); and emotional in the sense that the learners' perceptions of the social context are related to their emotions. Isohätälä et al. (2018) claim that notions as *socio-emotional processes* or *relational space* are attempts to capture the efforts of participants to sustain cohesive social interactions.

Building on these approaches to the role of emotions, our study seeks to add knowledge to argumentation studies by examining how preservice teachers build emotional tension while engaged in argumentation in the context of decision-making about the dilemma of omnivorous vs. vegetarian diets (VDs). This SSI context was chosen by considering the growing concerns on how to feed global population (Food and Agriculture Organization of the United Nations (FAO), 2009), the long-term effect of diets on health (International Agency for Research on Cancer, 2015), and the environmental impact of human nutrition. Although for decades the environmental focus has been on energy sources and uses, a growing area of research assesses the impact of diets on sustainability (Stehfest et al., 2009; Thompson et al., 2013; Tilman and Clark, 2014; Hyland et al., 2017) and on climate change. The Intergovernmental Panel on Climate Change (IPCC, 2019) has issued a special report, including a chapter on food security, which attributes about 21–37% of total greenhouse gas emissions to the food system. In order to reduce this impact, the report suggests that healthy and sustainable diets should be high in vegetables and low in animal products, such as meat, while acknowledging that dietary changes are guided by social, cultural, environmental, and traditional factors; and hence recognizing the influence of affective dimensions related to culture and traditions. It should be noted that environmental disruption and intensive livestock production are also identified as part of the causes involved in the cross-species transmission of the coronavirus in the coronavirus disease of 2019 (COVID-19) pandemic (Cui et al., 2019; IPBES, 2020); as Zheng-Li Shi, a leading expert on coronavirus diseases, claimed on her WeChat: “The 2019 novel coronavirus is a punishment by nature to humans' unsanitary life styles.”

Against this backdrop, the goal of our study is to examine in which ways emotional tension frames the construction of arguments about vegetarian vs. omnivorous diets (ODs) using preservice teachers. The research question is:

In which way does emotional tension act as a process of framing in an argumentative debate?

First, we discuss recent approaches to the role of emotions in argumentation; second, we present the data sources and methods; and third, we discuss the findings and their potential research and educational implications.

## Rationale: Emotions and Argumentation

Argumentation and decision-making about SSI deal with complex issues, demanding the consideration of different dimensions and drawing from multidisciplinary perspectives. In many cases, these arguments address open-ended dilemmas as they do not have a “best” solution that can meet all requirements from different dimensions. As Morin et al. (2014) pointed out, these open-ended issues bring out the complexities and uncertainties embedded in ill-structured problems, reflecting social representations and value systems. In arguments about SSI, a range of dimensions, besides scientific knowledge, such as values, ethical concerns, cultural habits, or emotions, are mobilized. Thus, SSI contexts can be suitable for the study of emotions as argumentative resources (Polo, 2014). We draw from the communication studies that define the framing as a rhetorical process through which communicators (consciously or unconsciously) construct a point of view to promote a particular interpretation of facts (Kuypers, 2009). Our goal is to explore how emotional tension may act as a process of framing in an argumentative debate. Under these premises, first, we discuss research about argumentation and emotions, and second, the relevance of discursive contexts in argumentation.

### Argumentation and Emotional Tension

Aristotle, in his *Rhetoric*, underlined that emotions are among the three means of persuasion (ethos, pathos, and logos), and nowadays it is generally acknowledged that argumentation involves both justification and persuasion (Jiménez-Aleixandre and Erduran, 2008). However, the relationship between argumentation and emotions has been, for years, an understudied issue. Emotions were absent in the seminal work of Toulmin (1958), one of the foundations for the study of argumentation in science education. This gap may be related to a bias in learning research toward the cognitive at the expenses of the affective (Baker et al., 2013b). Thus, Baker et al. (2013a) pose questions such as how do interpersonal relations, the circulation of emotions, and collaborative learning interrelate. In his pioneer work about emotions, Plantin (2011) questioned the antagonism between reason and emotion, with origins in the stoic philosophers, pointing out that they are inseparable. It should be noted that in the 17th century Baruch Spinoza (1677/2012) challenged the Cartesian mind-body dualism, suggesting a relationship between emotions and rational decisions, a challenge that caused his exclusion from the Jewish community. The relevance of Spinoza's ideas is being reappraised in the field of psychology of emotions (Brown and Stenner, 2001).

An increasing interest in the role of the emotions on the argumentative discourse has arisen, particularly in the French linguistic tradition, drawing from the work of Grize (1996) and his notion of schematization, a discursive representation of *discourse objects*. Within this tradition, there has been a distinction between *emotional*, the authentic psychological emotions the participants may feel, and *emotive*, what is discursively expressed. However, there is no evidence that expressed and felt emotions are actually different (Polo et al., 2017; Herman and Serafis, 2019), so we will use them interchangeably. According to Voloshinov (1986), the relationship between discourse and emotions is dialectical, and utterances imply both an evaluative stance toward interlocutors and an emotional positioning. By discourse, drawing from a sociolinguistic perspective, we mean not merely language in use, but language situated in ongoing sociocultural practices with histories, discourses, intertextual references, and social relationships of members of the relevant social group (Kelly, 2021).

It should be noted that the focus of our study is neither on determining participants' felt emotions, nor on the validity of their arguments, but rather on understanding how they mobilize the emotions as argumentative resources for argumentative purposes within the discourse, as proposed by Plantin (2011), and how, in doing so, they sustain socio-emotional interactions (Isohätälä et al., 2018). This entails methodological challenges as emotions can be present in multiple forms in the discourse, being frequently implicit (Herman and Serafis, 2019). Plantin (2011) suggested a need for moving beyond the lexical analysis in order to reach the implicit emotional component of the discourse through the inferences built on cultural stereotypes. We draw from his work about tension in argumentation (Plantin, 2019) to analyze the role of emotions in participants' argumentative exchanges in terms of what we call *emotional tension*. He characterized tension, from the point of view of argumentation, as “an operator showing that the speaker is highly involved in her speech and wants to share her commitments, that is, wants to persuade her audience” (Plantin, 2019, p. 348). Furthermore, he pointed out that “*Tension variation* is the defining feature of emotions in general—in discourse as in interactions” (ibidem, p. 362; Plantin's italics). Therefore, we suggest that *emotional tension* is an adequate term for the analysis of these interactions, as it was in agreement with Plantin (personal communication). The analytical framework and the coding categories derived from it are discussed in the “Methods” section.

Framing their work in collaborative learning, Baker et al. (2013a) addressed the interrelationships among emotions, interpersonal relations, and learning in their edited volume, with one section devoted to argumentation and emotion. Within it, Muller Mirza (2013) focused on the ways interlocutors in argumentative discussions may interpret or frame disagreements and conflicts, in other words on the conflictual dimension of argumentation. She examined how these conflicts may foster or hinder epistemic argumentation, suggesting elements for the design of a “thinking space,” which creates opportunities for the integration of identity and affective processes in argumentative practices. The relationships between epistemic, *what* participants are saying, and interpersonal, *how* they say it, dimensions of argumentation are the focus of Asterhan's (2013) chapter, also paying particular attention to different attitudes toward conflict resolution, which result in two types of argumentative discourse, consensus seeking and adversarial. Asterhan (2013, p. 255) pointed out how affective concerns “may divert students' attention away from the epistemic dimension of the conflict (a conflict between ideas) and heavily focus on the interpersonal dimension of the conflict (a conflict between persons).” This may result in argumentative discourses either void of the critical dimension, because the participants seek a quick consensus, or void of collaborative knowledge construction (adversarial). In the first case, disagreement with peers may be perceived as hampering positive relationships and acceptation. She suggested the interest of engaging students in the discussions that are both critical and co-constructive, otherwise they may experience difficulties in combining these dimensions. This balance, or lack thereof, is relevant for our study. Argumentative discourse is a sociocultural practice; Brown and Stenner (2001) discuss the significance of Spinoza's ideas for the studies about the social construction of emotions; for instance, the tension between “what might be characterized as ‘materiality' and ‘the work of thought”' (p. 98), where materiality corresponds to bodies and thought to minds. Personal emotional processes are also in play when engaging in argumentation; for instance, Richter and Maier (2017) examine how readers' prior beliefs may lead to a biased processing of conflicting information. The meaning of “beliefs” in the context of the task is discussed below.

The place of identity and emotions in argumentative learning has been addressed by Schwarz and Goldberg (2013). In line with Plantin's (2011) approach, they considered identity and emotions as resources in historical reasoning. Identity conflicts in historical argumentation of Spanish (Galician) high school students have been examined by López-Facal et al. (2015), showing how national identification influenced their arguments, which were different when the discourse object was Ireland from when it was the Basque Country, a historical national within Spain that was perceived as affecting their own national identity. In our study, participants' Galician identity, mostly implicit, is appealed to in their arguments as discussed in the findings. Although science issues have been considered “cold” and unaffected by emotions, science education research has shown the influence of motivational and affective factors (Darner, 2019). Darner, focusing on science denial, called for a recognition of science topics as emotionally laden, which is the case of dietary choices. In science education, Avraamidou (2020) discussed the process of forming a science identity, with an emphasis on recognition and emotions, and she suggested that emotions can offer a valuable lens for studying inequalities. In Hufnagel (2021), identity is a reference point for emotions. In her program of research about emotional sense-making in the context of climate change, emotions are conceived as evaluative mechanisms that indicate personal relevance and deep relationship to ideas or objects (Hufnagel, 2015). Her model for the analysis of emotions considered them as social and situated, pointing out that “emotions are not internal entities but a relationship to a specific event, experience, idea, and so forth” (Hufnagel, 2019, p. 156). For our study, the identity that matters is being Galician, on two dimensions: first, the evaluation of the impact of the adoption of a VD in Galician economy, where breeding is very important; second, on the consideration of eating meat as part of the cultural identity of participants.

Being relevant to our work, Isohätälä et al.'s (2018) study discussed about how student teachers, in the context of a teacher education course on environmental science, struck a balance between engaging in argumentation and sustaining socio-emotional processes favorable to it. They found that the groups sustained favorable socio-emotional processes, but mostly failed to engage in argumentation. The participants generally refrained from critical discussions, accepting each other's claims or conceding to divergent claims without argumentation; the challenging nature of argumentation may cause participants to attend to socio-emotional processes at the expense of cognitive ones. This is a question pointed out by Asterhan (2013) as discussed above. The authors suggested the need for more studies from authentic “messy” learning contexts.

As a summary, there is a growing interest in the intertwining of emotional and cognitive processes in argumentation, a body of research to which this study seeks to contribute. Our focus is on how emotional tension frames argumentative processes as it is one of the ways of exploring the role of emotions in argumentation.

### Discursive Context of Decision-Making

Jiménez-Aleixandre and Brocos (2018) suggested that argumentative operations and products are likely to differ depending on discursive contexts specific to pedagogical discursive practices, such as constructing and evaluating causal explanations, or making decisions, which is the one addressed in this study. This is an analytical frame conceived for research purposes, as in actual classroom settings these contexts may overlap. While in the construction and evaluation of *causal explanations* and models the aim is to choose the model that is best supported by evidence, *decision-making* is characterized by the use of evidence in order to make a decision or to choose a course of action. There is a set of common operations, for instance, using appropriate criteria for identifying and evaluating genuine evidence; identifying which evidence is valid and relevant for the issue at stake; considering multiple claims, theories, or options; or engaging with each other's ideas, supporting or challenging them. However, because cognitive and emotional processes are situated, other operations are particularly relevant in a given context. For instance, generating rebuttals is of relevance to eristic settings when two contrasted arguments, sometimes about emotionally charged issues, are opposed. However, in cooperative work in small groups, participants need to be able to conceptualize arguments different from their own, and a mark of quality may be the co-construction of arguments among several participants (Jiménez-Aleixandre et al., 2000). A distinction between contexts may be that, in decision-making, the discursive path can proceed from evidence to claims, as, for instance, in the study of Bravo-Torija and Jiménez-Aleixandre (2018); while in the evaluation of causal explanations the discussion proceeds, in many cases, from the alternative claims to the evidence supporting them (Jiménez-Aleixandre et al., 2000). In their work about shifts in epistemic status in argumentation, Jiménez-Aleixandre and Brocos (2018) suggested that in the context of developing explanations and models, the focus is rather on the individual learner whereas in decision-making the focus is on the participants in a social interaction. Furthermore, while plausibility is a relevant feature in evaluating explanations and models, in decision-making the focus is on acceptability “which indicates not only the degree of feasibility of the options considered, in light of the available evidence and previous ideas, but also their accordance with personal and social values” (Jiménez-Aleixandre and Brocos, 2018, p. 174–175). Thus, for instance, participants may believe or not that carrying out a specific option—as, in this study, the VD—it is individually or socially possible; and for doing so they have to take into account if the option is consistent or not consistent with other conceptions and values individually or socially accepted, such as those attached to different cultures and traditions in a given context. These beliefs in the plausibility of a given option may influence how they evaluate information (Richter and Maier, 2017), while their Galician identity is a reference point for emotions (Hufnagel, 2021), and may increase the emotional tension in this decision-making context.

## Data Sources and Methods

### Methodological Approach, Participants, and Learning Context

This study adopts a qualitative method approach, seeking to analyze educational case studies through expressions and actions in their local contexts (Denzin and Lincoln, 2013). Qualitative approaches are appropriate to study processes and evaluation practices (Creswell, 2013). It makes part of a wider study with 85 preservice primary teachers (PST). Studies on the challenges experienced by teachers for supporting their students' engagement in argumentation and SSI are limited, in comparison with the studies on students (Evagorou and Puig, 2017). In this paper, we analyze the arguments of a small group of four PST in which all participants agreed to be recorded. They were enrolled in a science education course taught by the first author, engaging in tasks about the evidence evaluation, criteria for strong arguments, and balanced diets; they sought the information about dimensions (environmental, ethical, nutritional, economic, or cultural) of diets, shared through a wiki, and constructed arguments in small groups, formed by them, about sustainable and healthy diets. The design of the teaching sequence is discussed in detail by Brocos and Jiménez-Aleixandre (2020). Ours is a bilingual context, where both co-official languages, Galician and Spanish, are used interchangeably and fully understood by all actors. The texts and debates have been translated into English by the authors.

Participants are identified with pseudonyms, beginning with the letter of their small group. In group B, there were three males and a female (Bea); their ages in years being 21 (Borja), 24 (Bea), 38 (Breixo), and 43 (Blas). While the ages of the two younger ones were in correspondence to the mean age of the whole class, the other two were significantly older.

Participants were asked to construct an argument about which diet they would consider to be better. The handout is reproduced in Annex 1 (see Supplementary Materials). In order to build that argument, they were directed to use a complex data set, consisting of their own selection of information, collected in a wiki, as well as five additional handouts elaborated by the researchers, one for each dimension (cultural, environmental, economic, ethical, and nutritional). These additional handouts were produced to ensure that for each dimension there is available information supporting different and even conflicting choices (Jiménez-Aleixandre et al., 2019).

### Data Collection and Rubric for Analysis

Data collection, through immersion of the second author as a participant observer, included participants' written products (individual pretest, portfolios, group final essay), video recording, and semi-structured interviews with three of the four participants (Blas, 32:54; Bea, 40:54; and Breixo, 48:3) 1 year later, after they had read the full transcription of the debate. For the purpose of this paper, the corpus comprises the video recording of a 90-min session devoted to construct the argument, their final essay, and the interviews.

Discourse was analyzed by using constant comparative analysis (Glaser and Strauss, 1967). The unit of analysis is the turn of speech, defined as each intervention by the participants. Turns were grouped into episodes and defined as one or several turns of speech related to the same topic or action (Gee, 2014); in this group we grouped turns into 10 episodes. Rubrics and coding categories emerged from the interaction of the theoretical frame with data in successive iterations. Transcriptions of the oral debate and the written essay were analyzed by both authors, initial repertoires of categories drawing from the literature were elaborated, and tentative codes were independently assigned to each unit.

Emotional tension was examined, primarily by analyzing its variations, and secondarily by considering the use of themes carrying affective weight. In order to analyze the variations in emotional tension, we constructed a repertoire, synthesized in Table 1, distributing the coding categories in High (H), Medium (M), and Low (L) emotional tension. The repertoire draws from:

- Plantin (2019) components of tension, such as *radicalization of arguments* (H), *interjections* (H), and *rhetorical questions* (H).
- Plantin (2011) categories for emotional positioning, which he considers an axis for the emotive construction of the discourse, representing the evaluation of a discourse object on a pleasant–unpleasant continuum, such as *life-death* (H) and *impact-consequences* (M). Also, we include one category for emotional intensity—a second axis for the emotive construction—*distance* (M), on a continuum from close to far, which may refer to a place or to the personal distance of participants.
- Andriessen et al.'s (2011) criteria for socio-cognitive tension and relaxation, such as *interrupting* (H), *taking stance* (M), *counterclaim* (M), *requesting clarification* (M), *compromise* (L), *focusing, and building* (L).
- Authors' categories, such as *summarizing* (L), *clarifying* (L), and *confirming, acknowledging contributions* from others (L).

**Table 1:** Coding categories: variations in emotional tension (drawing from Andriessen et al., 2011; Plantin, 2011, 2019).

**Tension**	**Categories**	**Characterization**	**Examples**
High emotional tension	Life-death	References to life are framed as positive, references to death as negative.	292 Blas: you can't die yourself to save the animals, mate.
	Interjections/exclamations	Exclamatory statements, cursing, rising intonation.	74 Blas: Damnit!
	Interruptions	Participants interrupting each other.	225 Breixo [interrupting] But, I mean, implicitly…
	Radicalization	All-or-nothing arguments rejecting compromises, excluding counter-discourse.	124 Blas: For instance, veganism seems aberrant to me.
	Rhetorical questions	Questions that both challenge and give no voice to the opponent; pretend to express a shared knowledge.	344 Blas: it has always been more ethical to hunt a rabbit in the wild than keeping 20,000 rabbits there locked up, all dejected, isn't it?
Medium emotional tension	Distance/closeness	Place distance/closeness, and people distance/closeness (identity).	35 Bea: if [vegetarian diet] is for many people, then… Galician economy would be damaged
	Taking stance	Expression of a position, claim.	46 Blas: It is a diet completely in favor of meat consumption […]
	Counterclaim	Opposition to a claim.	116 Breixo: No, lactovegetarian you don't eat meat.
	Requesting clarification	Asking about meaning/about evidence.	156 Breixo: Omnivorous diet. Arguments for it? Which are your data?
	Impact/Consequences	Desirability or not of the consequences (real or expected) of the situation.	26 Bea: But if everybody chooses the vegetarian option, it would affect Galicia negatively.
Low emotional tension/Negotiation	Seeking compromise, negotiating	Looking for a position that would be acceptable for both sides.	132 Bea: You can also be partially vegetarian.
	Summarizing	Recapitulating information or positions.	147 Blas: environmental impact [of meat diet] […] more greenhouse gases
	Focusing, Building	Directing debate to the task goals and adequate procedures	275 Breixo: Yes, I agree, but… we need to support it in argument.
	Clarifying	Explaining meanings or positions	154 Blas: Fish… maybe.
	Acknowledging contributions, confirming, agreeing	Recognizing or validating ideas and inputs.	218 Breixo: Fine, [what Blas said] then I write… meat industry would be reduced, and compensated with increase in agro-industry.

It should be noted that the distribution of categories for emotional tension in three levels is a simplification; on one hand, some categories are better seen as a continuum between increasing and decreasing tension. On the other hand, the significance of certain utterances is dependent on the task and the specific moment-to-moment interactions. The purpose is to capture the overall tension of each episode. Plantin (2011) identified the cultural conventions that emotionally frame the discourse. For instance, we can argue that conceptualizing an event as being close in terms of place, like it is the case with Galicia in this study, leads to stronger emotional response. Similarly, presenting a situation as going against widely recognized social identities (people closeness) may lead to an unpleasant emotional atmosphere. The categories for variations in emotional tension and their characterization are summarized in Table 1, with instances from our data.

The emotive framing of the issue, characterized through these categories, configures a certain emotional atmosphere for the debate, from higher tension, more eristic, to lower tension, associated with negotiation.

The use of themes carrying affective weight was analyzed by drawing from Plantin (2011), Polo (2014), and from the following two categories developed by Hufnagel (2015, 2019): *aboutness*, referring to the objects of emotion, and *type of feeling*. Kerbracht-Orecchioni (1980) discussed affective substantives and adjectives, which enunciate both features of the object as well as “une réaction emotionnelle du sujet parlant en face de cet object” (p. 84). Supplementary Table 1 summarizes the themes appealed to by each participant. The selected themes satisfied either of the following criteria: (a) three or more mentions; (b) strong intensity; or (c) pleasant–unpleasant positioning. On the intensity axis, the more frequent theme carrying emotive weight was “supplements,” with 23 mentions; the theme “pills” was mentioned in five occasions, and it was coded separately because of its semantic association with medicine and illness. Second in frequency is a cluster of “meat reduction,” with 11 mentions, opposed by the claim that VD implies no meat at all (7). Closeness of the participants evidenced, for instance, by the use of first and second person, and by direct references to Galicia, was also used.

On the emotive positioning axis, “ethical” has the highest frequency, 21 references, although, as discussed below in relation to episode four, in some cases it seems a ritual invocation rather than a deep reflection on the issue. Second and third in frequency are two clusters: the second, situated on the pleasant side, concerns “argument,” “evidence,” and “criteria,” with 19 references, expressing alignment with the norms for good arguments; the third, around “death,” “to kill,” and “slaughterhouse,” is located on the unpleasant side, with nine references.

## Data Analysis—Findings: Fluctuations of Emotional Tension

Analyzing the variations in emotional tension in the argumentative debate provided opportunities for a better understanding of the intertwining of cognitive and emotive processes. Before describing the findings, we summarize the relative participation of the four group members, which was uneven. There are 356 turns of speech in the session, 348 leaving out eight clarifications by the instructor and the researcher (first and second author). From these, Borja made only nine, while the other three had, respectively, 125 (Blas), 122 (Breixo), and 92 (Bea). Borja's utterances were barely substantive, asking questions about task procedures or expressing agreement; for this reason, the analysis emphasizes the conversation of the three members who contributed significantly.

### Fluctuation of Emotional Tension Across Episodes

We begin with an overview of the emotional tension across episodes. Table 2 summarizes the distribution of the session in episodes, the content or topics of each episode, and the number of H, M, and L tension turns in each one. As the number of turns is uneven, we provide the percentage in order to give an idea of the emotional climate in the episodes. It may be observed that, for 6 out of the 10 episodes, more turns—from 74.3 to 41.2%—are coded as L; in one episode, L and M are tied; in two episodes—second and ninth—M are more frequent; and, in the last episode, H dominates. In other words, tension tends to increase as the debate moves forward.

**Table 2:** Level of tension in each episode.

**Episode (turns)**	**Content**	**Coded turns**	**Emotional tension**	**# turns**	**%**
1 (1–48)	Agreeing on task goals	**43**	H	0	-
			M	14	32.68
			L	**29**	67.4
2 (49–87)	Discussing nutrition data	**39**	H	5	12.8
			M	**21**	53.8
			L	13	33.3
3 (88–105)	Social vs. personal choice	**17**	H	5	29.4
			M	5	29.4
			L	**7**	41.2
4 (106–146)	Proposing intermediate options: OR	**39**	H	8	20.5
			M	13	33.3
			L	**18**	46.2
5 (147–179)	What is your evidence?	**31**	H	3	9.7
			M	11	35.5
			L	**17**	54.8
6 (180–220)	Weighing economic impact of VD for Galicia	**39**	H	1	2.6
			M	9	23
			L	**29**	74.3
7 (221–247)	Negotiating OR option	**25**	H	7	28
			M	**9**	36
			L	**9**	36
8 (248–280)	Discussing ethical dimension	**23**	H	5	21.7
			M	7	30.5
			L	**11**	47.8
9 (281–337)	Disagreement OR vs. VD	**49**	H	14	28.6
			M	**19**	38.8
			L	16	32.6
10 (338–356)	Can OR be ethical?	**17**	H	**12**	70.6
			M	3	17.6
			L	2	11.8

*OD, Omnivorous Diet; OR, Omnivorous Reducing Meat; VD, Vegetarian Diet; VG, Vegan Diet. Coded turns, excluded the ones from the researchers and the inaudible or neutral. The bold values in column 5 represent the highest category in each episode*.

### Central and Supporting Arguments

There is a central argument running across the 10 episodes, which opposes the two options or clusters of options, OD and omnivorous reducing meat (OR) vs. vegetarian (VD) and vegan (VG) diets. OD was proposed and justified by Blas and Bea, with support from Borja; and VD by Breixo, who appealed to evidence pointing to the benefits of VD for health, the environment, and animal well-being. He sometimes defended VG, rather for the sake of the argument, considering that it was not his personal option, as he made explicit in the interview. The OD proposal, from episodes one to three, evolved to OR when Blas (112) suggested it.

The other five arguments are considered as secondary, supporting the central one. From these, the more frequent, in six episodes, dealt with the social vs. personal character of the choice, justifying OD on the basis that VD would not be adequate for a whole society; advanced by Bea as early as in turn 24, was then backed by Blas. The second in frequency, in four episodes, initiated by Breixo, argued that VG could be nutritionally adequate with the use of supplements, which was strongly opposed by Blas; then the argument evolved to the issue of whether supplements could be considered “food” or not, “natural” or not, and hence if such VG, involving supplements, should be socially promoted.

**Figure 1:**
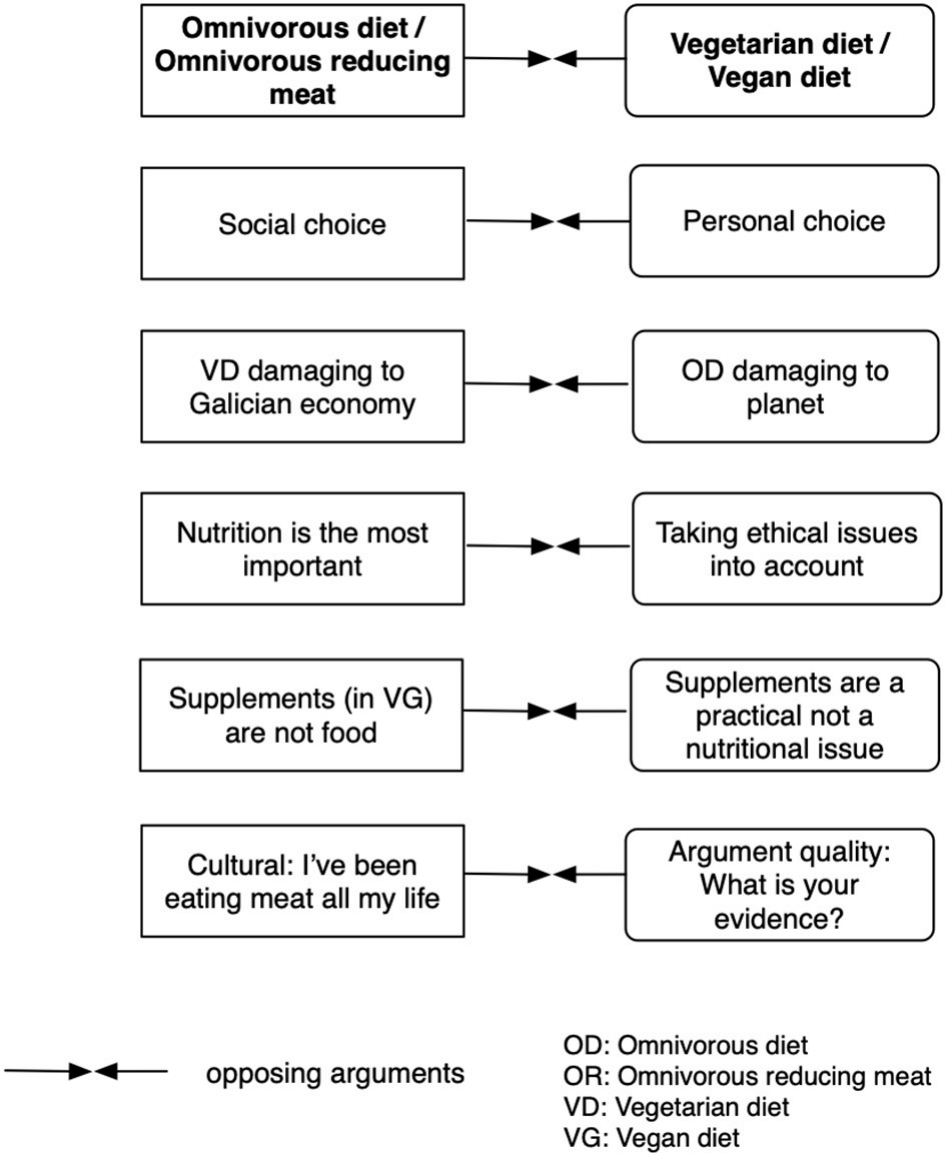
Main two arguments opposing omnivorous diet (OD)/omnivorous reducing meat (OR) to vegetarian diet (VD)/vegan diet (VG) and supporting arguments.

A third supporting argument was contextualized in Galicia—the autonomous region where the university is located—and it justified OD in the damages for Galician economy of a radical reduction of meat intakes. In the economy handout, part of the data set provided, there is information about the weight of breeding (66.6%) over agriculture (28.7%) in the livestock-farming complex in Galicia, as well as the relevance of the food industry in Galician exports. This argument was explicitly carried out in three episodes. The remaining two supporting arguments were not always explicit although they were underlying a great deal of the debate: the relevance accorded to nutrition over other dimensions in opposition to ethical concerns, and the cultural weight of OD and eating meat, in contrast with Breixo's insistence in requiring evidence for OR. Figure 1 represents the six arguments.

The transition from proposing OD to OR, in episode four, may illustrate how several argumentative lines and emotive moves are intertwined in the discourse of participants:

**Table:** 

108 Breixo: You, which diet… which diet seems more adequate? Which one would you defend?	L
109 Bea: Huh… At first glance I would defend the omnivorous one.	M
110 Breixo: Omnivorous. You? [to Blas]	L
111 Bea: [interrupting] But, wait, I would like to add…	H
112 Blas: Huh, omnivorous but with higher vegetarian presence, a lacto-vegetarian or something… a very occasional meat consumption…	L
113 Bea: [at the same time] Yes, sure, perhaps with higher meat reduction	L

114 Breixo: But, look, then it is omnivorous… look, lacto-vegetarian is not omnivorous.	M
115 Blas: Huh… okay, no, but…	M
116 Breixo: No, lacto-vegetarian you do not eat meat.	L
117 Bea: But with meat reduction.	M
118 Blas: [interrupting] No, you eat [sic] milk or… reducing meat, omnivorous diet, yes.	H
119 Breixo: Sure, what I think is…	-
120 Bea: With a… through an ethical and rational thinking and perhaps reducing the amount of meat in diets…	M
121 Breixo: But then… but then that is omnivorous.	L
122 Bea: Yes.	L
123 Breixo: You [to Blas] are not saying omnivorous.	M
124 Blas: No, but neither am I completely saying, I am not saying… for instance, veganism seems aberrant to me.	H

Breixo asked everyone to make explicit their choices, which should be implicitly justified in data about the impact of each diet in health and nutritional needs, as previously discussed. This prompted Bea and Blas to state their OD choice, and to propose a reduction of meat consumption, implicitly taking into account that nutritional data pointed to health risks of OD with a high meat intake. This argumentative move is interpreted as an offer of negotiation, seeking a consensus. However, in a rhetorical move, this OR diet was “sugarcoated” by Blas (112) under the term “lacto-vegetarian.” This originated an exchange about its meaning with Breixo, who appealed to the criterion of the presence or absence of meat. Furthermore, Bea (120) supported OR with another rhetorical move qualifying meat reduction as “ethical and rational thinking,” which is interpreted as introducing emotive tones. We distinguish these nominal appeals to ethics from actual debates about the ethical dimension, in terms of animals' well-being, which will be addressed later. As a summary, Bea and Blas central argument “OD is the more adequate” was supported in two ways: first, using the modified claim “omnivorous reducing the amount of meat”—which would implicitly address criticisms to health risks and environmental damage derived from a regular OD—later even labeled as “lacto-vegetarian”; and second, in a generic appeal to ethical thinking. In episode 4, low tension (L) predominates as it happens in six out of the first eight episodes.

A repeated supporting argument justified OD on the basis that VD would not be socially adequate. It was first advanced by Bea (26) in episode one, appealing to the impact of VD on Galician economy, coded as M, place closeness, and its relevance was acknowledged by Breixo.

26 Bea: Sure, because for instance… here [handout] it tells about Galician economy, does it? About diets huh… what happens? That for instance reading about ecology, vegetarian is better. I mean, it is the one less harming for the planet, that is what they say in this document. But if everybody would choose the vegetarian option, it would affect Galicia negatively. Then, it is not the same that we would decide a diet for all or personally.

Bea's argument had a double-edged claim that exemplifies the conflicting nature of SSI, which is a scientific question with social consequences. Rather than an explicit defense of OD, the claim was a criticism to VD, justified in the negative effects for Galician economy if VD becomes widely adopted; despite acknowledging that VD is better for the environment. This emotive framing has a strong place-closeness component. In episode three, the orientation changed from damage to economy toward the social impossibility of taking supplements: Bea's utterance (95) is coded as H, a rhetorical question:

95 Bea: Would you [to Breixo], for society, promote a diet that would require supplements? Rather than a balanced diet that would have food…

Although in episodes one and three there is a higher frequency of L utterances, the issue of supplements resurfaced again in episode seven in a heightened tone; in that episode, L and M are tied with nine turns each, and there are seven H turns:

**Table:** 

224 Bea: Huh… I believe that if you are going to recommend something, I mean, if it were a personal decision I wouldn't mind, in fact I sometimes take supplements, although I am not vegetarian, but…	M
225 Breixo [interrupting] But, I mean, implicitly…	H
226 Bea:…to promote it as something social, I wouldn't promote something that would need complements to be…	M
227 Breixo: Fine, but why? Because implicitly you are recognizing that it is a pain in the ass to deal with supplements… or why?	H
228 Bea: But not because it is a pain in the ass, but because the diet that I am recommending has deficits and needs from other	

things, from, er… the chemist or as the pharmacy or…(…)	H
235 Blas: [interrupting] Imagine, to tell people that from now on… imagine we are the ministry and we were in a banana republic… from those that we could impose what people eat, wouldn't be? Sure, to tell people that we are going to have a diet with… that they need to go to the pharmacy to buy supplements… it seems to me something…	H

After the debate about supplements, the focus of the social-personal dilemma shifted to the need for them, in a move overlapping VGs—which need supplements—with VDs—which do not. Breixo (227) criticized the justification, which he considered of a practical nature, not nutritional. Blas (235) heightened the emotional tension: first, comparing hypothetical recommendations to impositions of a banana republic and second, opposing food to products bought in pharmacies. It should be noted that the improbability of a mass adoption of VD was not discussed.

### Emotional Tension as a Process of Framing

In the participants' discourse, fluctuations in emotional tension interacted with the evaluation of evidence across a range of dimensions. We focus primarily on the building of a frame that oriented the consensus and the conclusions stated in their final essay, and secondarily on the use of themes carrying affective weight.

The emotive building of a frame through an interaction between emotional tension and evidence may be illustrated by the appeals to ethical issues. It represents a conflict between two norms that are difficult to reconcile: the consideration of meat as the standard diet, and ethical principles that would establish the undesirability of mistreating or killing animals. Ethics were first invoked by Bea in episode four, turn 120 (reproduced above), when she proposes a reduction in meat consumption. She further develops this issue later in the same episode:

**Table:** 

144 Bea: I am saying something varied, [eating] a little bit of everything. But with… with rationality, I mean without slaughterhouses that cause… excessive suffering to animals, to value the… the ethical dimension… a little bit of everything with rationality.	H
145 Blas… [interrupting] And intensive fisheries exploitation…	H
146 Bea: I… I mean, I don‘t know whether the best option would be the vegetarian one, but to me… in my view… fuck, I've been eating meat all my life and… I believe that if you do it with rationality and responsibility, it could be a good option for society [rising intonation on “society”].	H

**Figure 2:**
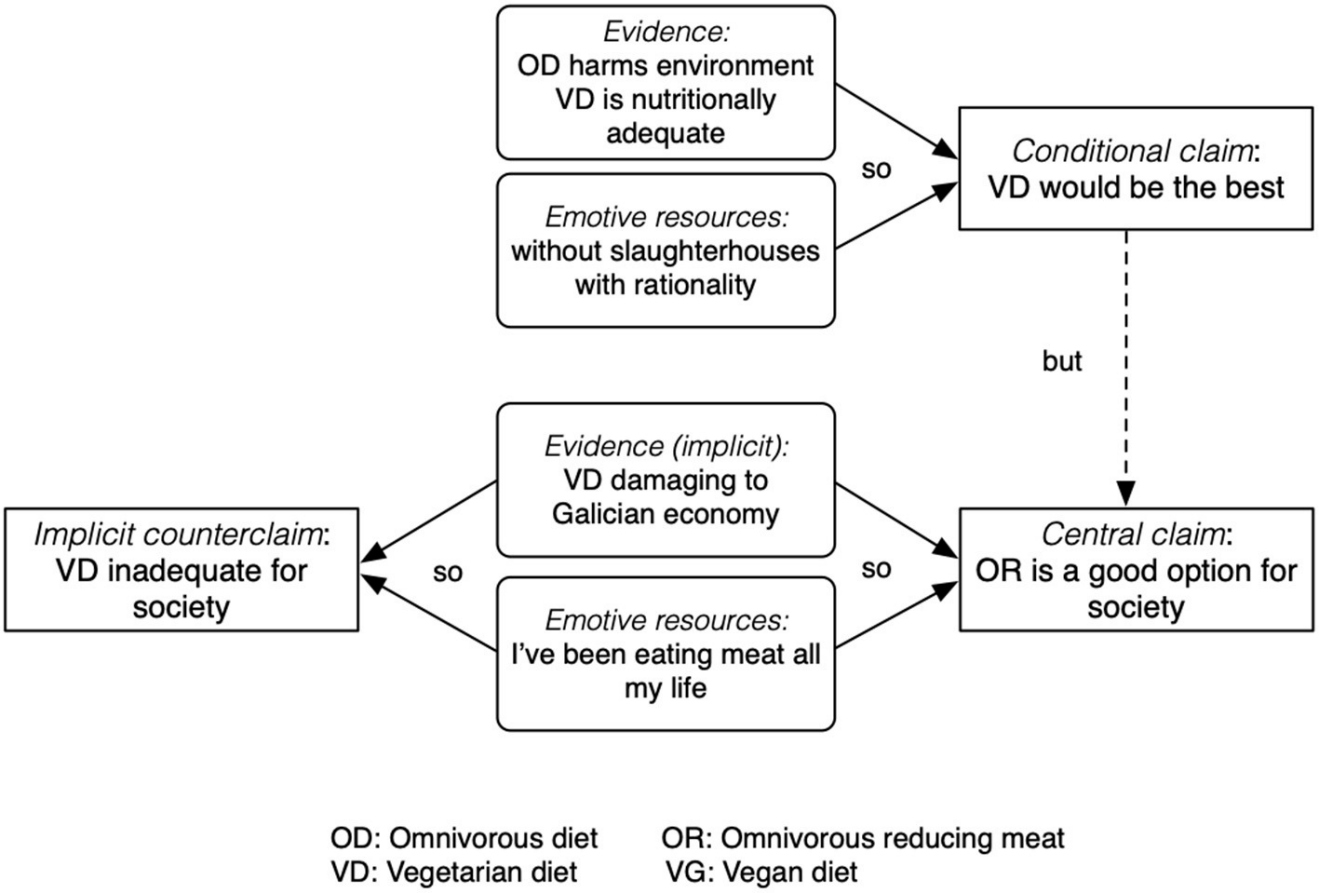
Bea's argument in episode four, combining evidence and emotive resources.

Bea and Blas' choice of OR was implicitly based on the evidence about environmental and nutritional impact of OD discussed in episodes 1–3. These pieces of evidence, as the one mentioned by Blas, are modulated by the interaction with values and emotions, such as the ethical dimension (Bea, 144), with a specific reference to animal suffering and to slaughterhouses, which carry an unpleasant emotive weight. She further continued (146) by laying out her argument: the choice of OR is based on the supporting argument “good option for society.” The justifications for this second claim were explicitly of cultural and emotional nature “I've been eating meat all my life,” and the claim is reinforced by the curse, the rising intonation, and implicitly by the anticipated impact of VD to Galician economy. All these utterances are coded as high tension (H). The intertwining of emotions and evidence in her argument are represented in Figure 2.

The conflict between two sets of values, ethical and cultural-emotional, is carried out to the last episodes, 8–10, when emotional tension reached its peak:

**Table:** 

250 Blas: Well, ethical dimension, which is ours… we talk about Peter Singer […] from two or several ways of feeding ourselves we should choose the one causing less harm, shouldn't we? Which is…	M
251 Breixo: Therefore the vegan one, right?	H
252 Blas: Sure, here it would opt for vegan ones, but huh… but we cannot open an ethical reason at the expense of one… of a nutritional argument.	M
253 Breixo: How?	M
256 Blas: Then we need to attend to nutrition, which is important. Sure, it is, why do we eat? Why do we have a diet? Why do we feed ourselves? In order to… gain something, whatever they are. Then, in this sense, I mean, we should contemplate ethics, but with priority to…	H
259 Bea:… it [omnivorous diet] would be missing a more ethical use that the one it has today and… I mean, it is not the most perfect option from an ethical perspective, but…	M
261 Bea: Sure, because to me is much more ethical to kill [lowers her voice] an animal in order to eat it and to use its skin for clothes than to kill it only to… to take leather, or things […] I believe that it is truly more ethical if you sacrifice an animal, make the most of its use completely…	H
267 Blas: Yes, yes, we have there a problem of ethics against nutrition.	L

In this excerpt from episode 8, Blas explicitly acknowledged the conflict between nutrition and ethics, claiming that they should give priority to nutrition, with a rhetorical question (256). Bea, in her efforts to build a supporting argument for an OR, continued to develop notions about more or less “ethical” ways of killing animals; it may be noted that she lowers her voice when saying “to kill” (261), arguably ashamed of acknowledging that eating implies killing.

In episode nine the debate was being framed in a life-death opposition, after Breixo challenged the other three members, who defended OR, to justify in which way that option would satisfy the ethical criteria for adequately treating animals:

**Table:** 

291 Breixo: Ok, ok, but ethically…? Why… why a reduced omnivorous diet? Because you are still eating animals.	M
292 Blas: Because… man, I eat some animals, but… you can't die yourself to save the animals, mate. That's… phew!	H
293 Breixo: But who is talking about dying…	H
294 [they speak simultaneously, inaudible]	–
295 Breixo: But it has been proved that you won't die if you don't eat animals. I mean, there are vegan people out there in the world.	H
296 Blas: An underfed human species, right? I want to belong to a hypertrophied human species, at least to a certain extent, right? [laughs]	H
297 Breixo: Ok, but come on, we have to argument properly.	M

Blas (292) claim set the issue as death either for humans or for non-human animals, framing it in a high emotional tone and driving the focus away from the ethical implications. He was implying that killing animals (in order to eat them) is unpleasant, but dying oneself is even worse.

The emotional tension keeps rising in the last episode, the 10th, in which 12 out of 17 turns are coded as H. Breixo accepted the OR option in order to build a consensus, although he still strived for developing an evidence-based argument, trying to meet quality criteria.

**Table:** 

338 Breixo: I… can accept it [your position]. But then I still don't… I still don't know what is leading you to make a reduction of meat consumption.	M
339 Bea: Okay, because of ethical reasons… So there is not…	L
340 Breixo: Ethical reasons, what? Because at the end the animal… you raise it to be… [he omits wat would probably be “killed”]	M
341 Bea: Well, but one thing is uh… to have a control of how this killing is and… all this process, and another thing is to do it massively as…	H
342 Breixo: And why is this more ethical?	H
343 Bea: Fuck!	H
345 Blas: Man, it has always been more ethical going to hunt a rabbit in the wild than keeping twenty thousand rabbits there locked up, all dejected, isn't it?	H
347 Breixo: I can tell you that breeding a rabbit… breeding rabbits to eat them goes against the basic interest of any animal, which is to carry on with living.	H

Bea and Blas, in their defense of the OD, made attempts to present the issue as a question of “killing animals ethically.” It may be interpreted as a way of avoiding the causal (unpleasant) implication of eating animals: in order to eat them, you have to kill them first.

**Figure 3:**
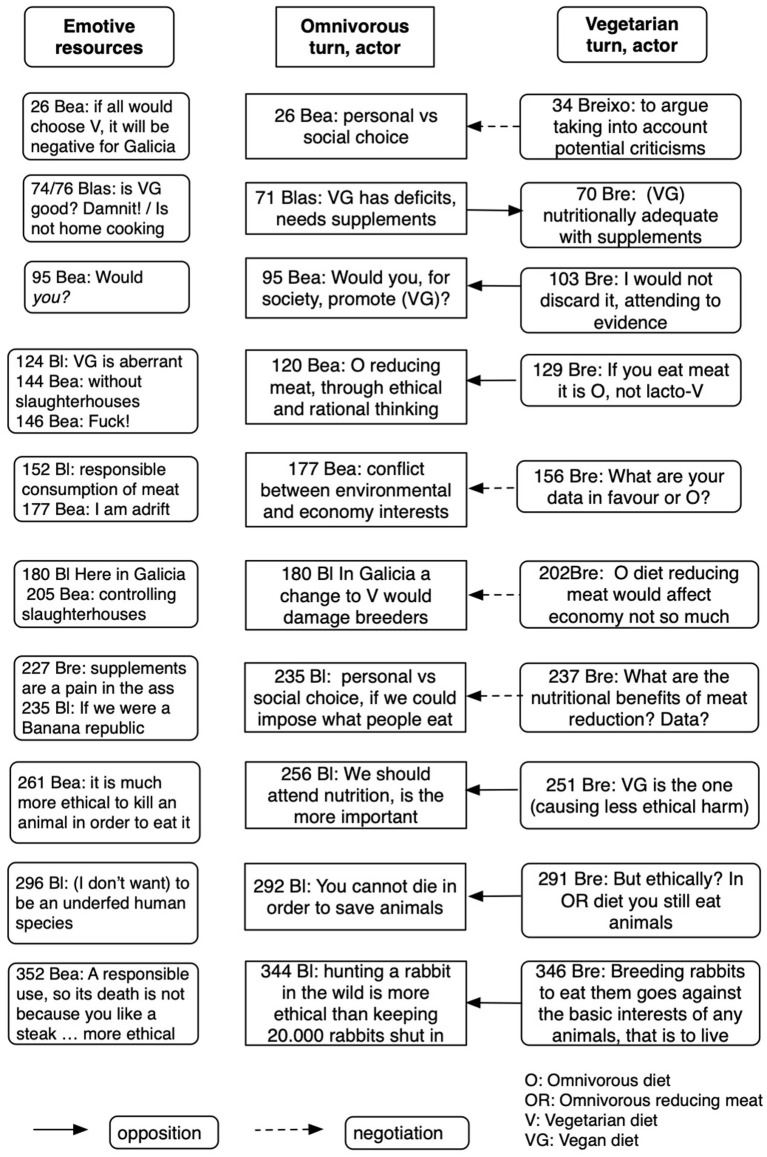
Emotive resources used in building an emotive frame through the 10 argumentative episodes opposing OD/OR to VD/VG diets.

In the last three episodes, particularly in the 10th, the argumentative exchanges are framed in a life-death emotive positioning. We interpret that, in this context, the emotional framing takes over the evaluation of evidence, which up to that point had been used by Breixo to support the vegetarian (or even vegan) options. Figure 3 represents the opposition between the two sides of the central argument across the 10 episodes, and how emotive resources are employed in building a frame that oriented the debate toward consensus—although, as discussed later, it was not an actual consensus, but sort of a forced one, arguably because of time constraints—and hence toward the decision stated in their final essay.

It may be noted that, as represented in Figure 3, participants appealed to emotive resources as soon as in the first episode: for instance, the negative (unpleasant) potential impact of a large-scale adoption of VDs for Galician economy, where cattle breeding has a greater weight than agriculture. This emotive positioning, related to a strong place closeness, resurfaced later in other episodes.

### How the Emotive Framing Oriented the Decision

As a summary of the process of negotiating a decision, the debate showed that the initial state was one of discursive opposition between OD and VD, and that the group's strategy was exploratory, collaborating, although with uneven participation, and focusing on the epistemic conflict between ideas, not on personal oppositions. However, contrarily to other small groups (Jiménez-Aleixandre and Brocos, 2017), we interpret that they did not reach a consensus, as displayed in the final turns of the last episode reproduced above. This was one of the questions posed in the interview: were disagreements resolved and how? To this, their responses differed:

Blas: Yes [they were solved]…by time pressure. Because it was necessary to produce a work, this facilitated reaching a consensus.
Bea: I don't remember [the disagreements]… It was difficult that Breixo would accept some of the ideas.
Breixo: No, they were not resolved… we didn't make ourselves understood to the others.

Furthermore, to the question about how was the decision reached, Breixo stated that it was necessary to deliver the work on time, adding:

Breixo: It has not only arguments, but also feelings and prejudices.

We interpret these responses in the light of the debates and the final decision about OR: from the two participants proposing OR, Bea stated that she did not remember the disagreements (even though she had the opportunity of reading the transcription), and Blas saw them solved and consensus reached, probably because the decision corresponded to his proposal. On the other hand, Breixo acknowledged that the differences had not been solved, in other words, that they wrote the essay without actually agreeing about the decision, even though he accepted to bring the debate to an end. Blas and Breixo pointed to time pressure −90 min of debate and previous work about the data set—and to the need for delivering an essay. Interestingly, Breixo added that the decision had been not only a matter of arguments, but also of “feelings and prejudices.” We may also note that in the interview the three student teachers identified the ethical dimension as the most relevant for them, something that is not clearly reflected in the debate, or in their decision.

We reproduce the two initial paragraphs, and also the last one, from their 1,000 word essay, which was submitted as their final decision resulting from the debate (emphasis in the original):

“In our group we agreed on a diet that we consider adequate to a person under normal health conditions, and that could even be promoted for the Galician population.
We propose a *low meat consumption diet*; an omnivorous diet that includes not only vegetables, but also small percentages of animal meat, selected in accordance with the criteria of a responsible consumption which seeks to reduce unnecessary animal suffering, ecological sustainability, to preserve economy, and to respect our own culture.”
(the four paragraphs discussing, respectively, the ethical, nutritional, environmental, and economic dimensions are not reproduced)
“We finally took into account the *cultural dimension* of the diet. We believe that this dimension should not be overlooked; we all are born and we live within a cultural context that conditions almost every of our day-to-day practices. (…) meat consumption plays an important role in the ‘traditional' Galician diet; therefore a proposal of change toward a meat-free diet would be very difficult since it would entail an important loss of a consolidated symbolic expression, which is also a part of our heritage. On the other hand, it would be feasible to propose a reduction of meat consumption, without drastically altering its cultural relevance.”

Emotions were mobilized in the written arguments, which show that they are not specific of oral contexts, although the emotional tension was toned down in comparison with the oral debates. Their final decision of an OD with low meat consumption was also the most frequent in the class: 10 out of the 20 small groups chose it in their essays. In this group, we interpret that the emotional tension framed the debate, which was essential in orienting the decision toward a diet that would be emotionally acceptable for all participants as discussed below.

## Discussion and Conclusions

This study examines how emotional tension acts as a process of framing in an argumentative debate about diets, and how this emotive framing drives the orientation toward the decision of an OD with meat reduction.

First, about the productivity of the argumentative process in comparison with the previous studies, the findings indicate that participants attended to the epistemic dimension of the dilemma, systematically discussing pieces of evidence related to each of the five dimensions involved: environmental impact of diets (episodes 1 and 5), nutrition (episodes 2–4), economy (episode 6), ethical issues involved in diets (episodes 7–10), and cultural dimensions (across several episodes). They did so while sustaining an exploratory discourse without interpersonal conflicts. Thus, they achieved, to a certain extent, productive discourse, avoiding the pitfalls pointed out by Asterhan (2013), of either excessive confrontation or lack of critical discussion. The participants in our study, like the ones in Isohätälä et al. (2018), maintained favorable socio-emotional processes. However, while Isohätälä et al. (2018) found that they mostly failed to engage in argumentation, the participants in our study did engage in it, although their evidence evaluation was not carried up to their final decision in a fully coherent way. We interpret that, while participants made extensive use of emotive resources, these were oriented toward the *discourse objects* (Grize, 1996), in other words, toward the diets, rather than toward other participants: they never had a personal target, which contributed to sustain a favorable affective climate. Cultural background may have played a role in a different way in which Galician (Spanish) student teachers' engaged in argumentation, in comparison with their Finnish counterparts in Isohätälä et al. (2018) study. This issue would need to be further explored in international comparative research.

Second, appeals to emotions and to evidence were deeply intertwined in the arguments of participants. This is reflected in the findings in several ways, for instance, in the range of themes employed in the emotive construction of the discourse, as summarized in Supplementary Table 1; particularly in the positioning axis. Participants framed the debate about diets on a life-death opposition, and developed meanings for “ethical” ways of eating, breeding, and even of killing. As Hufnagel (2015, 2019) has shown, emotional sense-making and meaning-making are related to the use of emotions as evaluative mechanisms, and this use points to personal relevance and deep relationships to ideas or objects (of discourse, we would add). A second instance is the content of the supporting arguments, represented in Figure 1, as, for example, the focus on social choices, the anticipated damages of VD to Galician economy, which has strong closeness for participants; or the cultural weight of family, and of traditional diets. A third instance are the arguments reproduced in the excerpts, revealing a combination of appeals to evidence and of emotional tension. Our results indicate that the construction of an argument and the construction of an emotive position are deeply connected, a finding coherent with Polo et al.'s (2013) findings.

Third, the findings suggest that arguments' quality, according to structural criteria, can be compatible with the integration of emotive resources. For instance, the written essay of the group is an example of integration of several lines of reasoning, articulating evidence, and values (Jiménez-Aleixandre and Brocos, 2017). Bea's argument in episode 4, represented in Figure 2, is a valuable argument from a structural point of view. However, in that argument, as in other cases, emotional tension is used to “reduce” the weight of the ethical considerations about ODs, be it OD or OR.

Fourth, the findings are coherent with our previous proposal (Jiménez-Aleixandre and Brocos, 2018) about the differences between discursive contexts of argumentation, in particular about the focus on acceptability in decision-making contexts, which is different from plausibility in the evaluation of causal explanations. For instance, while in arguments about potential explanations for the yellow color of farm chickens (Jiménez-Aleixandre et al., 2000), the students evaluate if it is plausible that the cause is heredity or eating yellow feed, in this study the debate is about the *acceptability* of VDs, particularly for its social implementation.

Finally, the analysis shows that the emotional tension built by two participants, Bea and Blas, was successful in achieving an emotive framing, which influenced the dynamics of argument construction in the group. In the central argument OD vs. VD, the benefits—attested by the pieces of evidence—of VD were weighed against what was implicitly perceived as threatening for lifestyles. Thus, in their essay, both in the first and last paragraphs, they focus on the feasibility of promoting a diet that would be acceptable for the Galician society, without challenging “a consolidated symbolic expression, which is also a part of our heritage.” In the diets' dilemma, more dimensions were emotively framed as negative (unpleasant) than as positive (pleasant), which arguably had consequences for the final decision.

We suggest that the analysis in terms of emotive framing, which is an original contribution of our study, may be a fruitful approach for sophisticated studies of argumentation about SSI. Future lines that we plan to explore through fine-grained analysis of the contribution of emotive resources are the relationships between the mobilization of emotions, emotional tension, and the participants' perceptions of their own agency. Educational implications are, for instance, the interest of designing argumentation tasks that specifically take into account this emotive dimension, which may lead to a deeper student engagement and personal agency. This is relevant for SSI and in particular for the question of sustainable diets as it depends largely on personal decisions rather than on institutional responsibility. Our findings suggest that reasoning is not performed in a neutral space, but rather is emotionally and personally motivated. On one hand, this could make more difficult the epistemic construction of arguments, but on the other hand, emotional implication could be an asset for engaging in urgent issues, as environment deterioration and climate change. As Brown and Stenner (2001) pointed out, discussing Spinoza, “Cartesianism, in setting us against nature, sets us against ourselves” (p. 87), a sentence that we read as an anticipation of the One Health concept–human health is inseparable from animal and plant's health. There is a need for humans to grasp these connections and set us for nature, rather than against it.

## Data Availability Statement

The raw data supporting the conclusions of this article will be made available by the authors, without undue reservation.

## Ethics Statement

Ethical review and approval was not required for the study on human participants in accordance with the local legislation and institutional requirements. The patients/participants provided their written informed consent to participate in this study.

## Author Contributions

All authors listed have made a substantial, direct and intellectual contribution to the work, and approved it for publication.

## Conflict of Interest

The authors declare that the research was conducted in the absence of any commercial or financial relationships that could be construed as a potential conflict of interest.
