# Supplementary material for: Emotional Tension as a Frame for Argumentation and Decision-Making: Vegetarian vs. Omnivorous Diets
Source: Front Psychol. 2021 Jun 8;12:662141. doi: 10.3389/fpsyg.2021.662141 (PMC8217629; doi:10.3389/fpsyg.2021.662141)
Supplement: Supplementary file 1 [file Table_1.docx]

Annex 1. Task handout

**Building an argument about diets**

1. **The task** consists of producing an argument about the dilemma of dietary choice working in small groups. The goal is to reach consensus within the group about what diet is the best choice (conclusion). It may be noted the diversity of possible choices: vegan diet, vegetarian diet, omnivorous with meat, omnivorous with fish, meat free days (e.g. Ghent’s Thursday Veggie Day), etc. The conclusion must be supported by data (evidence), drawn from the information handouts (documents 1 to 5), the wiki (online) and previous knowledge (justification), which may help to relate data and evidence. Your argument can take values into account.

2. **Dimensions:** Notice that this dilemma involves different dimensions (cultural- personal, ecological, ethic, nutritional, socioeconomic), so you can study the information handouts (data) and discuss partial questions separately such as: what is best from a cultural/personal point of view? What is best for the environment and the Earth? What is best for economy and society? What is best from an ethic point of view? What is best for health and nutrition? The answers to these questions (partial conclusions) can function as different lines of reasoning that can be integrated into a final conclusion.

3. **Criteria** for strong arguments:

- Taking the available evidence (data, information) into account.

- Stating the conclusion clearly.

- Specifying which pieces of evidence support the conclusion and which ones refute or criticize the choices rejected.

- Indicating what theories or knowledge were used to relate data and conclusions (justifications). If that is the case, specify what values support the conclusion.

- Integrating as many dimensions as possible in the argument.

4. **Writing a persuasive argument:** Once consensus is reached, you must write down your argument in order to persuade, for instance, another student of the faculty that your choice is the best one.

Table S1. Analysis of themes by categories for the two axes in the emotive framing of discourse (from Plantin, 2011, Polo, 2014, Hufnagel, 2019).

| **Axes** | **Categories** | **Bea N:** 52 E/92 | **Blas N:** 56 E/125 | **Breixo N:** 68 E/122 |
| --- | --- | --- | --- | --- |
| **Intensity** (stronger / weaker)  s / w | Agency, control | For society (3) - s  Control breed (2)  We decide  I don't know (2) | Banana republic - s  Impose a diet - s | Remove cattle  It is unclear |
|  | Distance - place | Close - Galicia (3)  Far -Australia (3) | Close - Galicia (3) |  |
|  | Distance - people  Ownership | Personal/social (7) s  Would you? (5) - s  For whom? (8)  To me (4)  All my life - s | Personal / social  You (3)  Our (diet)  Why do we eat? - s | I would / not (2)  You: (9)  What do we want?  You don't convince me (2) -s |
|  | Aboutness | Supplements (9)  Better diet (3)  Balanced diet (2)  Agree  Disagree  Meat reduction (3)  Part vegetarian (2) | Supplements (6) - s  Pills (5) - s  Better diet: (2)  Balanced diet  Agree (3)  Conflict (2)  Meat reduction (6)  Nutrition first (3) s | Supplements (8)  Agree (3)  Disagree (2)  Meat reduction (2)  VD = no meat (7) - s |
| **Positioning** (pleasant / unpleasant)  p / u | Life-death | Slaughterhouse (2)u  To kill animals (2)u  Death - u  Suffering - u  Caged / free (2)  Sacrifice - u  Fatten up - u | You cannot die to save animals - u  Kill in the wild - p  Underfed human - u  Caged/ dejected (2)  Look a cow in the eye vs pills - p | Who is talking about dying?  You don't die for not eating animals  Animals' interest to live vs eating them (2) |
|  | Conformity with norms | Ethical (7) - p  Rational (4) - p  Responsible (2) - p  Argument  Adequacy  Deficit (3) - u  Natural is best - p  Culture (proverb) -p  Food /not food | Ethical (9) - p  Responsible - p  Evidence - p  Adequacy  Deficit (4) - u  Natural is best - p  Home cook - p | Ethical (5) - p  Realistic  Practical (4)  Evidence (8) - p  Argument (7) - p  Criteria (2) - p  Natural ≠ good  Critique proverb - u  Food /not food |
|  | Impact  Consequences | On economy - u  On the planet - u  Threat - u | On economy (3) - u  Wreak planet - u  On environment (2)  Overfishing - u | On economy - u  On resources (2) - u |
|  | Type of feeling | Harm (2) - u  Fuck! (3) - u  Aha! - p  Adrift - u  Pain in the ass - u  [laugh] | Harm (3) - u  Fuck! / Damnit! (2)  Aha! (2) - p  Aberrant - u  liking: 23  [laugh] (3) | Harm (3) - u  Fuck! / Damnit! (2)  Rats!  Funny  Pain in the ass - u  [huff & puff] |
